# Retraction Note: Effect of apoptosis in neural stem cells treated with sevoflurane

**DOI:** 10.1186/s12871-024-02448-2

**Published:** 2024-02-23

**Authors:** Jianlei Qiu, Pengcai Shi, Wude Mao, Yuyi Zhao, Wenshuai Liu, Yuelan Wang

**Affiliations:** 1 Department of Anesthesiology, Dezhou People’s Hospital, Dezhou, Shandong China; 2 School of Medicine, Shandong University, Ji’nan, Shandong China; 3 Department of Anesthesiology, Shandong Provincial Qianfoshan Hospital, Ji’nan, Shandong China; 4 Department of Anesthesiology, Jiaozhou Central Hospital of Qingdao, Qingdao, Shandong China; 5 Department of Emergency, Dezhou People’s Hospital, Dezhou, Shandong China

**Retraction Note** **:** **BMC Anesthesiol 15, 25 (2015)**

**https://doi.org/10.1186/s12871-015-0018-8**

The Editor retracted this article because of concerns regarding a number of figures presented in this work. These concerns call into question the integrity of the data. An investigation conducted after its publication discovered several similarities both between images in this work and with images in [1], namely:

- The top band in Figure 3A (labelled GABAA R-S) and the top band in Figure 4A (labelled Bcl-2-S);
- The bottom band in Figure 4A (labelled GADPH) and the bottom band in Figure 4 (labelled GADPH) in [1];
- The bottom band in Figure 5A (labelled GADPH) and the bottom band in Figure 6C (labelled GADPH) in [1];

The Editor therefore no longer has confidence in the integrity of the research presented in this article.

Jianlei Qiu, Yuelan Wang, Wude Mao, and Yuyi Zhao disagree with the retraction. The remaining authors did not respond to the correspondence from the publisher about this retraction.
